# Supplementary material for: Axonal mechanisms mediating γ-aminobutyric acid receptor type A (GABA-A) inhibition of striatal dopamine release
Source: eLife. 2020 Sep 1;9:e55729. doi: 10.7554/eLife.55729 (PMC7462615; doi:10.7554/eLife.55729)
Supplement: Transparent reporting form [file elife-55729-transrepform.pdf]

eLIFE
1st Floor
24 Hills Road
Cambridge CB2 1JP, UK
P 01223 855340
W elifesciences.org
T @elife

eLife's transparent reporting form

Sample-size estimation

- You should state whether an appropriate sample size was computed when the study was being designed
- You should state the statistical method of sample size computation and any required assumptions
- If no explicit power analysis was used, you should describe how you decided what sample (replicate) size (number) to use

Please outline where this information can be found within the submission (e.g., sections or figure legends), or explain why this information doesn't apply to your submission:

Although we report the exact p-values to three decimal places for all of our results, sample size was not computed when the study was designed. However, we performed post hoc power analysis (using GPower 3.1) for comparisons that reached statistical significance ($p<0.05$). Most of our positive findings fall above a 0.8 power value. However, in one case in which we found statistically significant values ($p<0.05$), we found that statistical power fell below 0.8. The significant difference ($p=0.008$) between the width of the axonal and somatic action potentials (Fig.1D) shows a power of 0.629.

Replicates

- You should report how often each experiment was performed
- You should include a definition of biological versus technical replication
- The data obtained should be provided and sufficient information should be provided to indicate the number of independent biological and/or technical replicates
- If you encountered any outliers, you should describe how these were handled
- Criteria for exclusion/inclusion of data should be clearly stated
- High-throughput sequence data should be uploaded before submission, with a private link for reviewers provided (these are available from both GEO and ArrayExpress)

Please outline where this information can be found within the submission (e.g., sections or figure legends), or explain why this information doesn't apply to your submission:

Only experiments in which all solutions could be applied are included and outliers were not omitted under any conditions. Where possible, all data from individual cells are shown. The exception was with time course figures where only averaged data were shown for clarity. However, the raw data from each averaged time-course is included in the transparency file of each figure. At least 3 animals were tested per condition.

Statistical reporting

- Statistical analysis methods should be described and justified
- Raw data should be presented in figures whenever informative to do so (typically when N per group is less than 10)
- For each experiment, you should identify the statistical tests used, exact values of N, definitions of center, methods of multiple test correction, and dispersion and

precision measures (e.g., mean, median, SD, SEM, confidence intervals; and, for the major substantive results, a measure of effect size (e.g., Pearson's r, Cohen's d)
- Report exact p-values wherever possible alongside the summary statistics and 95% confidence intervals. These should be reported for all key questions and not only when the p-value is less than 0.05.

Please outline where this information can be found within the submission (e.g., sections or figure legends), or explain why this information doesn't apply to your submission:

Statistical significance was determined in 2 group comparisons by two-tailed Mann-Whitney U-test or Wilcoxon signed-rank test (paired comparisons) and in more than 2 groups comparisons by one-way ANOVAs or one-way repeated measures ANOVAs (paired comparisons) followed by the Bonferroni or Sidak post hoc test. Means are shown plus/minus SEM, medians are shown with 25/75 confidence interval box plots and 95/5 whiskers, otherwise raw data are always shown. This information is clearly stated in the Results section as well as in the materials and methods. A table describing all statistical tests described in the paper is appended to this worksheet.

(For large datasets, or papers with a very large number of statistical tests, you may upload a single table file with tests, Ns, etc., with reference to sections in the manuscript.)

Group allocation
- Indicate how samples were allocated into experimental groups (in the case of clinical studies, please specify allocation to treatment method); if randomization was used, please also state if restricted randomization was applied
- Indicate if masking was used during group allocation, data collection and/or data analysis

Please outline where this information can be found within the submission (e.g., sections or figure legends), or explain why this information doesn't apply to your submission:

There was no group allocation

Additional data files ("source data")
- We encourage you to upload relevant additional data files, such as numerical data that are represented as a graph in a figure, or as a summary table
- Where provided, these should be in the most useful format, and they can be uploaded as "Source data" files linked to a main figure or table
- Include model definition files including the full list of parameters used
- Include code used for data analysis (e.g., R, MatLab)
- Avoid stating that data files are "available upon request"

Please indicate the figures or tables for which source data files have been provided:

All Figures have the source data provided in excel spreadsheets

eLife Sciences Publications, Ltd is a limited liability non-profit non-stock corporation incorporated in the State of Delaware, USA, with company number 5030732, and is registered in the UK with company number FC030576 and branch number BR015634 at the address 1st Floor, 24 Hills Road, Cambridge CB2 1JP | August 2014

Comparison	Figure	n	Statistical Test	p-value	power
Firing Rate axon v. soma	1B	axon (41), soma (10)	Mann-Whitney U	0.298	N/A
Spike width axon v. soma	1D	axon (27), soma (10)	Mann-Whitney U	0.008	0.63
AP Threshold axon v. soma	1E	axon (26), soma (10)	Mann-Whitney U	<0.0001	0.99
Recording distance vs Interspike Slope, one-phase decay against a linear fit	1G	27	Comparison of Fits F test	<0.0001	0.99
Input Resistance main v. striatal axon	1J	striatal axon (74), main axon (28)	Mann-Whitney U	<0.0001	0.99
Avg. Interspike Voltage soma v. main axon	1K	soma (10), striatal axon (74), main axon (21)	Dunn's multiple comarison following Kruskal-Wallis Test	0.0317	0.96
Avg. Interspike Voltage main axon vs. striatal axon	1K	""	""	0.865	N/A
Avg. Interspike Voltage soma v. striatal axon	1K	""	""	0.0007	0.98
Baseline v Picrotoxin, peak depolarization following GABA puff	2C	9	Paired t-test, 2-tailed	0.0003	0.99
Low Cl- v. high Cl-, peak depolarization following GABA puff	2D	low Cl (10), High Cl (5)	Unparied t-test, 2-tailed	0.0008	0.92
Difference between Avg. interspike voltage and reversal of muscimol-evoked depolarization	2G	15	Paired t-test, 2-tailed	<0.0001	0.99
Main effect of GABA on peak dopamine release	3F	7	2-Way Repeated Measures ANOVA	0.004	0.99
Difference in slopes between GABA- and Depolarization-mediated shortening of APs	4D	GABA (14), Depolarization (15)	Comparison of Fits F test	0.047	0.93
Difference in slopes between GABA- and Depolarization-mediated slowing of AP rate of rise	4E	GABA (13), Depolarization (8)	Comparison of Fits F test	0.564	N/A
Difference in slopes between GABA- and Depolarization-mediated changes in AP peak and AP rate of rise	4F	GABA (11), Depolarization (8)	Comparison of Fits F test	<0.0001	0.99

Comparison between muscimol and baseline for long distance imaging	5C	5	Sidak's test following a 2-Way RM ANOVA	0.34	0.9
Comparison between muscimol and baseline for short distance imaging, caudal	5F	5	Sidak's test following a 2-Way RM ANOVA	0.95	N/A
Comparison between muscimol and baseline for short distance imaging, rostral	5I	8	Sidak's test following a 2-Way RM ANOVA	0.61	N/A
Effect of diazepam on dopamine release comapred to baseline	6B	6	Bonferroni's test following a 1-Way RM ANOVA	0.044	0.86
Effect of GABA antagonists on optically evoked dopamine release, measured with voltammetry, over baseline	Supplement 6, B	10	Paired t-test, 2-tailed	0.015	0.89
Effect of GABA antagonists on electrcially evoked dopamine release, measured with dLight over baseline	Supplement 6, D	11	Paired t-test, 2-tailed	0.101	N/A
